# Downregulation of Npas4 in parvalbumin interneurons and cognitive deficits after neonatal NMDA receptor blockade: relevance for schizophrenia

**DOI:** 10.1038/s41398-019-0436-3

**Published:** 2019-02-21

**Authors:** Ryan Shepard, Kelsey Heslin, Payton Hagerdorn, Laurence Coutellier

**Affiliations:** 1 Department of Psychology, The Ohio State University, 225 Psychology Building, 1835 Neil Avenue, Columbus, Ohio 43210 USA; 2 Department of Neuroscience, The Ohio State University, Wexner Medical Center, 333W. 10th Ave, Columbus, OH 43210 USA; 3 Present Address: Moores Cancer Center, University California, San Diego, 3855 Health Sciences Dr, La Jolla, CA 92093 USA; 4 Present Address: Interdisciplinary Graduate Program in Neuroscience, University of Iowa, Iowa City, IA 52242 USA

## Abstract

Dysfunction of prefrontal parvalbumin (PV+) interneurons has been linked with severe cognitive deficits as observed in several neurodevelopmental disorders including schizophrenia. However, whether a specific aspect of PV+ neurons deregulation, or a specific molecular mechanism within PV+ neurons is responsible for cognitive deficits and other behavioral impairments remain to be determined. Here, we induced cognitive deficits and altered the prefrontal PV system in mice by exposing them neonatally to the NMDA receptor antagonist ketamine. We observed that the cognitive deficits and hyperactivity induced by neonatal ketamine were associated with a downregulation of Npas4 expression specifically in PV+ neurons. To determine whether Npas4 downregulation-induced dysfunction of PV+ neurons could be a molecular contributor to the cognitive and behavioral impairments reported after neonatal ketamine, we used a transgenic Cre-Lox approach. Reduced Npas4 expression within PV+ neurons replicates deficits in short-term memory observed after neonatal ketamine, but does not reproduce disturbances in general activity. Our data show for the first time that the brain-specific transcription factor Npas4 may be an important contributor to PV+ neurons dysfunction in neurodevelopmental disorders, and thereby could contribute to the cognitive deficits observed in diseases characterized by abnormal functioning of PV+ neurons such as schizophrenia. These findings provide a potential novel therapeutic target to rescue the cognitive impairments of schizophrenia that remain to date unresponsive to treatments.

## Introduction

Disruption of the prefrontal GABAergic system has been reported in neurodevelopmental disorders marked by cognitive deficits. Interestingly, in many of these disorders, including schizophrenia and autism, the function and/or expression of parvalbumin-expressing (PV+) interneurons in the prefrontal cortex (PFC) is altered. Analysis of postmortem human brain tissues revealed decreased number of PV+ neurons in the PFC of patients affected by autism^1^. Likewise, the PFC of schizophrenia patients is marked by reduced expression of the calcium-binding protein PV and of GAD67, the rate-limiting enzyme necessary for the synthesis of GABA^2,3^. PV+ interneurons provide strong inhibition to pyramidal cells. They play a key role in generating gamma oscillations that have been implicated in proper cognitive functioning^4,5^. These findings and many others (e.g. ref. ^6^) support the important role played by prefrontal PV+ interneurons in cognitive impairments observed in a range of psychiatric diseases^7^.

Many studies robustly describe how disruption of PV+ neurons functioning lead to cognitive deficits in rodent models (e.g. refs. ^8–10^). However, whether specific molecular mechanisms within PV+ interneurons contribute to their dysfunction and to cognitive deficits remain unclear. Recently, studies have highlighted the potential involvement of the brain-specific transcription factor Npas4 to the molecular abnormalities and symptoms of schizophrenia. Npas4 is expressed in an activity-dependent manner in excitatory neurons, but also in all inhibitory neurons subtypes, including somatostatin-, vasoactive intestinal polypeptide-expressing and PV-expressing cells^11^. Npas4 regulates excitatory/inhibitory balance by promoting increased excitation onto inhibitory neurons, thereby lowering overall circuit activity^11^. Evidence from our laboratory indicate that Npas4 deficiency in mice leads to severe behavioral impairments that are reminiscent of those observed in schizophrenia, including social, sensori-motor gating and cognitive deficits, and hyperactivity^12^. We further reported that, in mice, Npas4 regulates the adolescent development of the prefrontal PV system^13^. We observed that developmental, as opposed to adult, downregulation of Npas4 is sufficient to induce impairments in cognitive flexibility and social behavior^13–16^, symptoms often observed in schizophrenia and autistic patients (e.g. refs. ^17–19^). A recent report supported our conclusions that Npas4 could be a contributor to the molecular and behavioral abnormalities seen in neurodevelopmental disorders characterized by abnormal functioning of the prefrontal PV system. Alachkar and collaborators^20^ showed that mice with prenatally dysregulated one-carbon metabolism, a known risk factor for schizophrenia, display schizophrenia-like behavioral deficits and have reduced Npas4 mRNA levels in their PFC. More importantly, their results in this mouse model were corroborated with human postmortem tissue of schizophrenia patients, which also showed downregulation of Npas4 in the frontal cortex.

The goal of the present study is to further investigate the potential role of Npas4 in prefrontal PV+ neurons deregulation and behavioral impairments as seen in some neurodevelopmental disorders. Specifically, we aimed to determine whether Npas4 downregulation could contribute to prefrontal PV+ neurons dysfunction and thereby could be a molecular mechanism underlying cognitive deficits in neurodevelopmental disorders. We first used a developmental mouse model of schizophrenia to induce dysfunction of prefrontal PV+ interneurons and behavioral anomalies, and to assess changes in Npas4 expression within this context. Here, we particularly aim to characterize the scope of Alachkar’s findings and investigate whether prefrontal downregulation of Npas4 is a generalized characteristic in rodent-based models of schizophrenia. We also intent to expand this previous report by showing that changes in Npas4 expression are cell type-specific and by proposing for the first time that Npas4 downregulation is specific to PV+ neurons and thereby could be an intracellular molecular contributor to their dysfunction. Finally, we used a genetic approach to assess the extent to which Npas4-dependent PV+ neurons dysfunction is sufficient to replicate the behavioral and cognitive impairments observed in our developmental mouse model of schizophrenia.

## Materials and methods

### Animals

All experiments were conducted in accordance with protocols approved by the Institutional Animal Care and Use Committee of The Ohio State University and were performed based on the National Institutes of Health Guide for the Care and Use of Laboratory Animals. All mice had access to food and water ad libitum, and were maintained on a 12-h reverse light/dark cycle.

For the neonatal ketamine exposure treatment, gestating C57BL/6J female mice were obtained from Jackson Laboratories (Bar Harbor, ME, USA). At birth (postnatal day PND1), male pups from each litter were divided into two experimental groups: a vehicle-injected group and a ketamine-injected group (KET). To avoid litter effects, no more than two pups from the same litter were used for our experiments.

For PV+ neurons-specific Npas4 conditional knockdown mice, Npas4 homozygous Flox mice (originally obtained from Dr. Greenberg laboratory, Harvard University, USA, and currently bred in our laboratory) were bred with PV:Cre mice (B6;129P2-Pvalbtm1(cre)Arbr/J, Jackson Laboratory, Maine, USA). Both lines were backcrossed to a C57Bl/6 background. After breeding for the F2 generation, mice were genotyped for the presence of the flox alleles, and for the presence or absence of Cre. Male mice of two different genotypes were used: PV:Cre^+^Npas4^fl/fl^ and PV:Cre^−^Npas4^fl/fl^ (control). Double immunofluorescent staining for PV (guinea pig anti-PV, 1:500—Synaptic Systems, Goettingen, Germany) and Npas4 (rabbit anti-Npas4 primary antibody, 1:2000—gift from Prof Greenberg, Harvard University, USA) was used to verify PV+ neurons-specific downregulation of Npas4 in PV:Cre^+^Npas4^fl/fl^ mice. Because we are specifically interested in the PFC, we counted the number of PV+ cells expressing Npas4 in three prefrontal sections from four adult male mice of each genotype using the optical fractionator method and the StereoInvestigator software from MBF Bioscience (Williston, VT, USA). The size of the counting frame, number of sites counted and dissector thickness were adjusted to maintain a mean coefficient of error ≤ 0.1.

All pups were weaned at PND21 and housed in cages containing 3–4 same-sex mice. Mice were left undisturbed until adulthood (aside from regular cage cleaning) when behavioral testing was conducted (starting at PND85–95) followed by brain collection.

### Neonatal ketamine exposure

Following the protocol of Jeevakumar et al.^21^, pups from the KET were given one daily subcutaneous injection of 30 mg/kg ketamine (Sigma, St. Louis, MO, USA) on PND 7, 9, and 11, while control vehicle-injected pups (VEH) received injections of 0.9% saline at the same time points. The injection of ketamine between PND7 and 11 induced a reduced body weight gain throughout this period (two-way ANOVA showing a treatment effect *F* = 13.68; *p* = 0.0003). Mice almost recovered by the time they reached adolescence (PND28; body weight VEH > KET; *p* = 0.057) and fully recovered by the time they reached adulthood (PND66; body weight VEH vs. KET *p* = 0.748).

### Behavioral analyses

All behavioral tests were conducted in adult male mice. All mice were habituated to experimenter handling for the 3 days prior to the start of behavioral testing for 1 min per day. The behavioral assays were chosen to cover multiple behavioral domains including general activity, emotional regulation (i.e. anxiety and despair), and cognitive functions. All behavioral tests were conducted during the active (dark) phase of the light cycle and were video recorded under dim white light conditions and scored by either a trained experimenter blind to subject group, or by using Ethovision XT 11.5 (Noldus, The Netherlands). Following the completion of all behavioral assays, brains were collected for molecular analyses of the PFC, including Western Blot and immunofluorescent analyses (3–4 animals per group).

### Behavioral testing in mice exposed to neonatal ketamine

VEH and KET mice were tested in three behavioral assays. Mice were first tested for their locomotor activity and anxiety-like behaviors in the open field test (OFT). Twenty-four hours later, they were tested in the object recognition task (ORT) to assess cognitive functions, and 48-h later, they were tested in the forced swim test (FST) to measure their depressive-like behaviors. A total of *n* = 8 VEH and *n* = 9 KET mice were tested.

#### Open field test

Mice were individually placed in a corner of a 40 × 40 cm unfamiliar square arena with opaque walls under dim white light conditions. They were allowed to freely explore the entire arena for a period of 10 min. Behaviors were recorded using an overhead camera and videos were scored using the tracking software Ethovision XT. Novelty-induced locomotor activity was measured using the total distance traveled (cm), and distance traveled (cm) near the walls and in the center of the arena as variables; anxiety-like behavior was measured using the time spent in the center (s) as variable.

#### Object recognition task

The protocol was adapted from Jeevakumar et al.^21^. On the first day, mice were allowed to habituate to the testing arena for 10 min. The following day, mice were exposed to four distinct novel objects in the testing arena for a 5-min learning phase. Following the learning phase, subjects were removed from the arena for a 2-min inter-trial-interval (ITI) while the arena and objects were cleaned with 70% ethanol. Immediately following the ITI, subjects were introduced back into the arena containing one familiar object from the learning phase in a new location and one novel object, and were allowed 5 min of exploration. Novel objects and locations were counterbalanced between groups. Time spent sniffing each object was manually scored from video recordings with sniffing defined as the subject’s nose making contact with the object, or being directed at the object within 1 cm. Discrimination ratios for the test phase were calculated by subtracting time spent sniffing the familiar object from time spent sniffing the novel object, divided by total sniffing time.

#### Forced swim test

Mice were individually placed in a glass cylinder (height: 30 cm; diameter: 15 cm) filled with water (24–25 °C) for 6 min. After the test, mice were placed back in their home cage on a heated pad until dry. Behaviors were recorded using a video camera to allow off-line scoring for latency to first immobility and total time spent immobile (in seconds). Mice were considered immobile when floating motionlessly or making only those movements necessary to keep its head above water.

### Behavioral testing in mice with Npas4 knockdown in PV+ neurons

PV:Cre^+^Npas4^fl/fl^ and PV:Cre^−^Npas4^fl/fl^ adult male mice were tested in five behavioral assays. They were first tested in the elevated-plus maze (EPM) test to measure anxiety-like behaviors. After a 24-h delay, they were tested in the Y-maze test to assess for spontaneous alternation, a measure of working memory. They then followed the same behavioral schedule as described above: OFT, ORT, and FST. However, to distinguish between short-term and long-term object recognition memory, mice were tested a second time in the ORT with an ITI of 24 h. A new unfamiliar object was used. Again, mice were allowed to explore for a 5-min period. A total of *n* = 7–8 PV:Cre^−^Npas4^fl/fl^ and *n* = 8–9 PV:Cre^+^Npas4^fl/fl^ mice were tested.

#### Elevated plus maze test

The apparatus consists of two open and two closed arms (33 × 6 cm) made out of white plastic and elevated about 55 cm from the floor. The closed arms feature 15 cm-tall grey plastic walls. Each mouse was placed individually in the central square of the maze with their head directed toward a closed arm. They were allowed to explore the entire apparatus for a period of 5 min. Each trial was video recorded and videos were analyzed using Ethovision XT. The total locomotion (cm), total arm entries, time in open arm (seconds), and number of entries in the open arm were analyzed. A video from a PV:Cre^+^Npas4^fl/fl^ mouse was corrupted and could not be analyzed, lowering *n* to 8.

#### Y maze test

Assessment of spontaneous alternation was performed as previously described^14^. Briefly, the apparatus consists of a gray plastic maze formed by three arms so as to form a Y shape (arm length: 40 cm; width: 8 cm; height: 15 cm). Each animal was placed in the apparatus for 5 min. Each trial was videotaped using an overhead camera and an experimenter blind to the genotype of the mice scored the succession of arm entries. Alternation was defined as successive entry into the three arms on overlapping triplet sets. Percent alternation was calculated as the ratio of actual alternations to possible alternations multiplied by 100.

### Western Blot analysis

Western Blot analysis was conducted in VEH/KET mice. Brains were collected 4 h after the FST, and immediately frozen on dry ice and stored at −80 °C. We chose a 4-h time point post-FST to avoid for potential confound due to molecular changes induced by acute exposure to FST. The PFC was dissected on dry ice in a cold room maintained at −15 °C and then processed as described previously^22^. The following antibodies were used: anti-parvalbumin (rabbit anti-PV, 1:500—ABCam, MA, USA) and anti-GAD67 (rabbit anti-GAD67 1:1000—Thermo Fischer Scientific, MA, USA). Signal was visualized using Clarity Western ECL Substrates with an HRP-conjugated secondary antibody for digital imaging (BioRad, CA, USA). Signal intensity was measured using the Image J Studio software whereby the mean gray value of each band of interest was measured and subtracted from the background value. The ratio of each protein of interest band value over the loading control (β-actin) value was then calculated.

### Immunofluorescent analysis

Brains were collected 90 min after the FST to count the total number of PV+ cells, the number of PV+ cells expressing Npas4, and the number of perisomatic PV-boutons^23^. The 90-min post-FST time point was chosen based on previous findings showing that protein level of Npas4 is highly expressed in neurons 60–90 min after a stimulus^24^. Briefly, animals were perfused with 4% cold paraformaldehyde (PFA) at baseline. Brains were removed and kept in 4% PFA at 4 °C overnight before storage in a sucrose solution (30% sucrose). Brains were frozen on dry ice and sectioned at 50 μm using a cryostat so as to obtain three sets of sections containing the PFC. Free-floating sections containing the PFC were treated for immunofluorescence staining using PV primary antibody (guinea pig anti-PV 1:500—Synaptic Systems, Goettingen, Germany) and Npas4 primary antibody (rabbit anti-Npas4, 1:2000—generously provided by Prof. Greenberg, Harvard University). Unbiased stereological quantitative analysis of the number of cells expressing PV and Npas4 in the PFC, and the number of non-PV somata surrounded by PV-boutons (as described previously^23^), was completed using the optical fractionator method and the StereoInvestigator software from MBF Bioscience (Williston, VT, USA) by an experimenter blind to the group of the animals. Regions of interest were outlined under low magnification (×5), and cells were counted at high magnification (×63 in oil immersion) in three sections per animal, with a section evaluation interval of 3. Counting criteria were determined to maintain a mean coefficient of error ≤ 0.1 for each region.

### Data analysis

Data were analyzed using Prism 5.01 (GraphPad Software Inc., CA, USA) using unpaired Student *t*-tests to compare experimental groups (KET or PV:Cre^+^Npas4^fl/fl^) to their respective controls (VEH or PV:Cre^−^Npas4^fl/fl^). For locomotor activity in the OFT, repeated measures ANOVAs were used with time bins as the repeated variable. Finally, correlation between the number of PV+ somata and the number of perisomatic PV-boutons in KET and VEH mice was calculated using the Pearson correlation coefficient. Statistical significance was set at *p* ≤ 0.05. Sample sizes per groups were chosen based on common standards in behavioral neuroscience and on power analyses using on our previously published data^12,13^. For molecular analyses (including Western Blot and immunofluorescent analyses) *n* = 3–4 per group is well-established. To confirm that sample sizes per groups were sufficient, a power analysis was conducted for each endpoint. Statistical powers (1-β) between 0.89 and 0.99 were calculated with effect sizes >1, supporting a sufficient *n* per group was used. Each experiment was conducted once in the groups of the stated sizes (*n*). All data are presented as the mean ± the standard error of the mean (SEM).

## Results

### Neonatal ketamine exposure increases activity in a novel environment and induces cognitive deficits

**Fig. 1:**
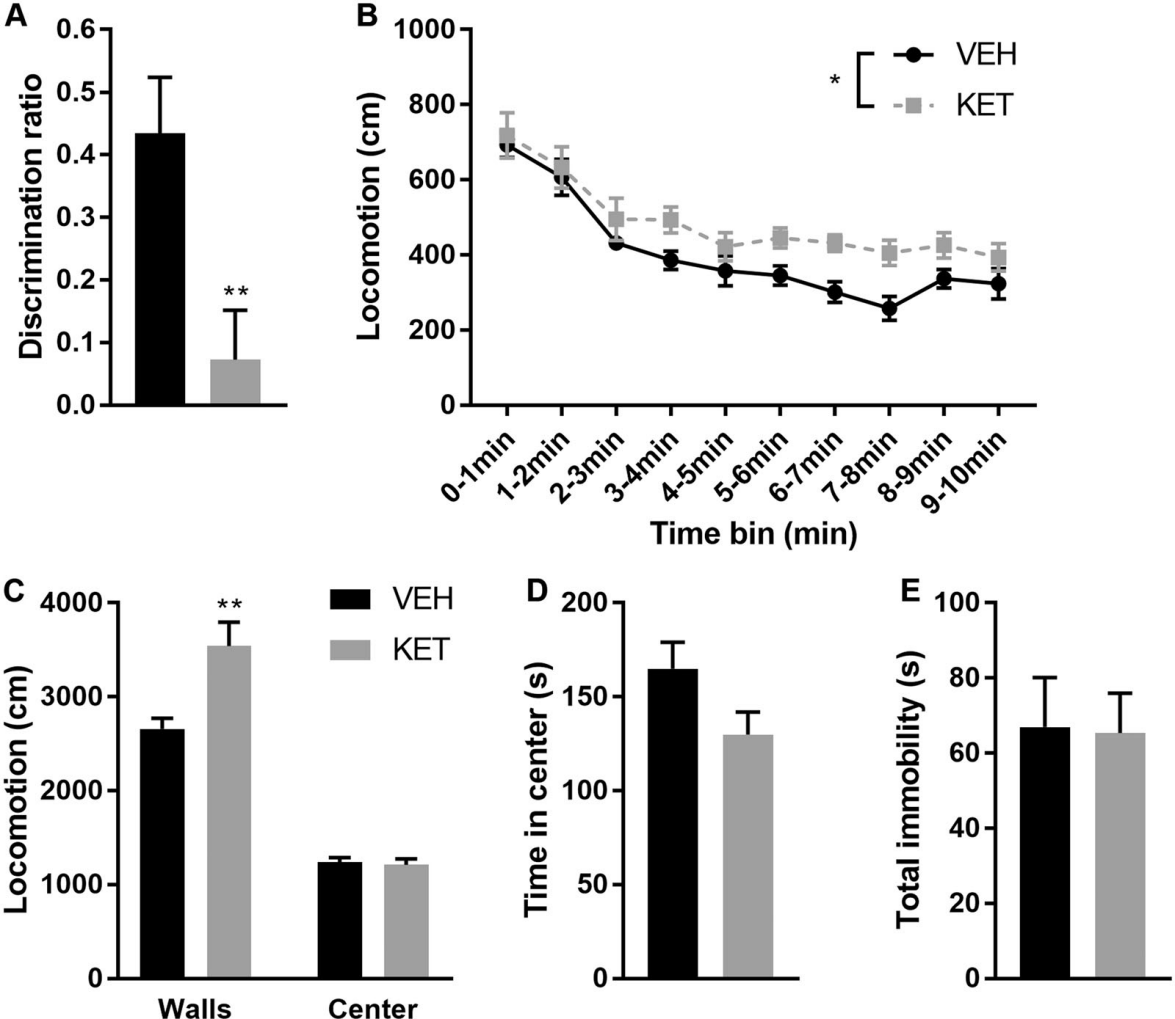
Mice neonatally exposed to the NMDA receptor antagonist ketamine (KET mice) display cognitive deficits and are hyperactive. **a** KET mice have impaired short-term object memory as measured by the discrimination ratio in the object recognition test (unpaired *t*-test: *t*_15_ = 3.044; *p* = 0.008). **b** KET mice have increased locomotor activity in the 10-min open-field test (two-way ANOVA with repeated measures: time bin effect *F*_9_,_135_ = 26.59; *p* < 0.0001; VEH vs. KET *F*_1_,_15_ = 8.005; *p* = 0.013). **c** The increased locomotor activity observed in KET mice is specific to the walls of the arena (unpaired *t*-test: *t*_15_ = 3.025; *p* = 0.008). **d** KET mice do not display increased anxiety-like behaviors as measured by the time spent in the center of the open-field (*p* > 0.05). **e** KET mice do not show despair-like behaviors in the forced swim test (*p* > 0.05). ^**^p ≤ 0.01; ^*^p ≤ 0.05; VEH *n* = 8; KET *n* = 9

We used a developmental model of NMDA receptor dysfunction to assess for specific changes in Npas4 expression in the PFC within the context of cognitive deficits associated with dysfunction of the prefrontal PV system. We first aimed at verifying behavioral alterations induced by neonatal exposure to ketamine. Prior studies using the NMDA receptor antagonists MK801 or ketamine in rodents have reported long-lasting cognitive deficits without significant changes in motor functions^21,25^. We similarly observed that KET mice displayed cognitive impairments in the ORT, as shown by a discrimination ratio significantly lower than VEH mice (*t*_15_ = 3.044; *p* = 0.008—Fig. 1a). This effect was not due to differences in learning or motivation to perform the task since the total time spent sniffing the objects during the learning and testing phases was not different between KET and VEH mice (*p* > 0.05). Unexpectedly, KET mice exhibited novelty-induced hyperactivity, indicated by a higher level of locomotion throughout the 10 min of the OFT (repeated measure two-way ANOVA: treatment effect *F*_1,15_ = 8.005; *p* = 0.013; time bin effect *F*_9,135_ = 26.59; *p* < 0.0001—Fig. 1b). Further analyses of activity patterns in the OFT showed that KET mice were more active near the walls of the arena (thigmotaxis behaviors; *t*_15_ = 3.025; *p* = 0.008) but not in the center (Fig. 1c). Finally, we observed no significant changes in emotional behaviors induced by neonatal ketamine exposure: the time spent in the center of the OFT (*p* > 0.05—Fig. 1d), as well as the time spent immobile in the FST (*p* > 0.05—Fig. 1e) were not affected by the treatment.

### Neonatal ketamine exposure induces abnormal prefrontal PV and Npas4 expression

**Fig. 2:**
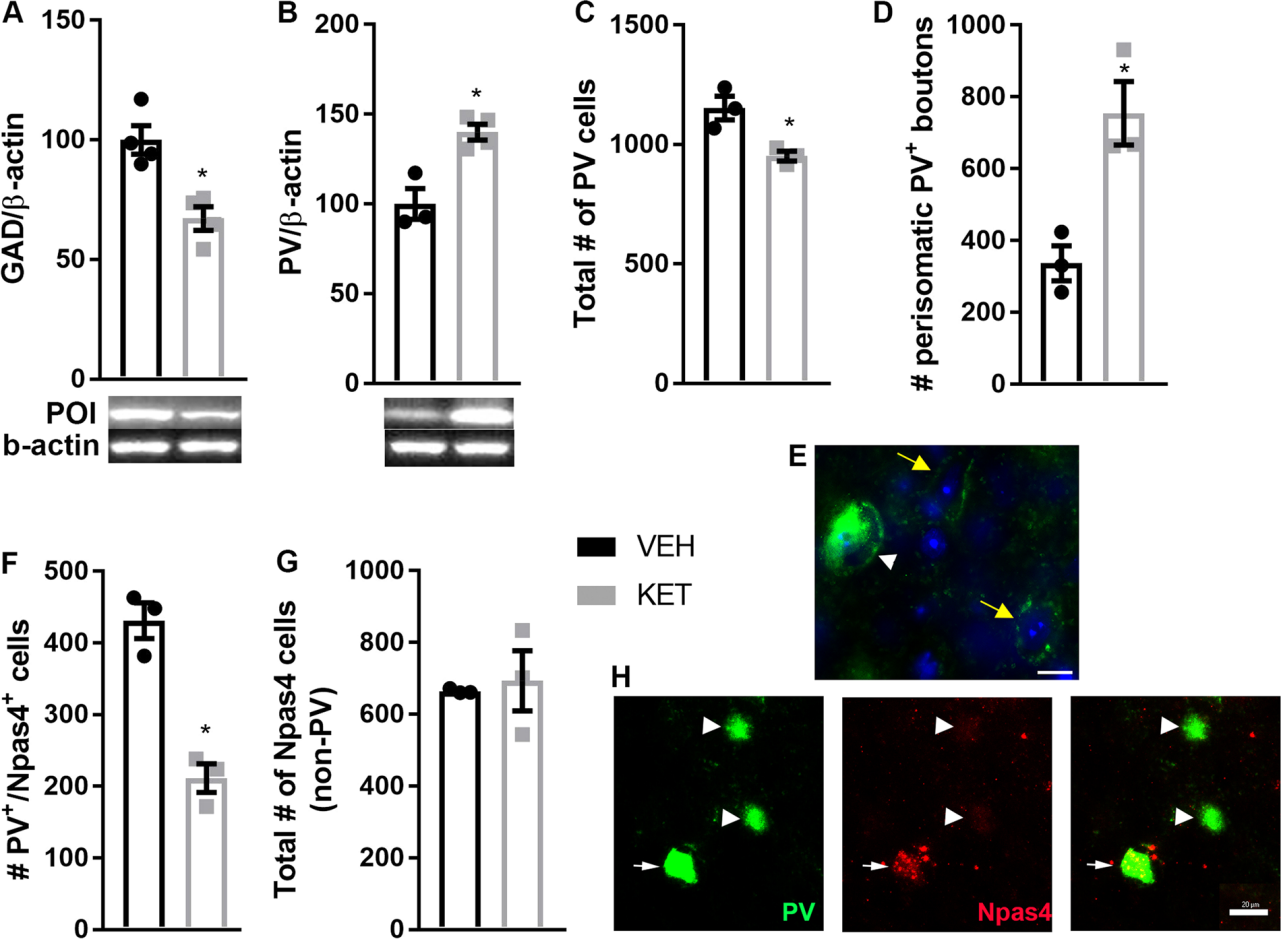
Mice neonatally exposed to the NMDA receptor antagonist ketamine (KET mice) show downregulation of Npas4 specifically in PV-I of the PFC. **a**, **b** Adult KET mice have decreased protein levels of GAD67 (*t*_6_ = 4.266; *p* = 0.005) and increased protein levels of PV (unpaired *t*-test: *t*_5_ = 4.466; *p* = 0.007) in the PFC when compared to VEH control mice. **c** KET mice have less PV+ somata in their PFC compared to VEH control mice (*t*_4_ = 3.779; *p* = 0.019). **d** KET mice have more PV+ boutons in their PFC compared to VEH control mice (*t*_4_ = 4.142; *p* = 0.014). **e** Representative picture of PV+ somata (white arrow head) and PV+ boutons (yellow arrows); PV is green, DAPI is blue. **f** KET mice have less PV+ cells expressing Npas4 (*t*_4_ = 6.871; *p* = 0.002), but (**g**) no change in the number of Npas4-expressing cells outside PV+ neurons (*p* > 0.05). **h** Representative pictures of immunofluorescence staining for PV (green) and Npas4 (red). Arrow points to a double stained cell (PV+ neurons expressing Npas4), while arrowheads point toward PV+ neurons not expressing Npas4. ^*^p ≤ 0.05; VEH *n* = 3–4; KET *n* = 4; POI = protein of interest. Scale bar = 20 µm

Multiple evidence points toward disruption of the prefrontal PV system after developmental exposure to NMDA receptor antagonist. Not only the number of neurons expressing PV is reduced, but also synaptic properties of prefrontal PV+ neurons are affected^21,26,27^. Our data further support disruption of the prefrontal GABAergic system after neonatal exposure to ketamine. First, KET mice displayed lower expression of GAD67 (*t*_6_ = 4.26; *p* = 0.005—Fig. 2a). We then observed that reduced number of PV+ neurons in the PFC of KET mice has previously reported (*t*_4_ = 3.78; *p* = 0.019—Fig. 2c). Surprisingly, western blot analysis of the total level of PV protein in the PFC indicated higher PV in KET mice (*t*_5_ = 4.46; *p* = 0.007—Fig. 2b). To try to reconcile the higher total level of PV expression despite reduced number of PV+ neurons in KET mice, we conducted further analyses of PV expression. Particularly, we counted the number of perisomatic PV-boutons (Fig. 2e) to determine whether the increased level of total PV expression could be explained by increased PV expression at the terminals of PV+ neurons in KET mice. We observed that KET mice have more PV-boutons (*t*_4_ = 4.14; *p* = 0.014—Fig. 2d). We also found a negative correlation between the number of PV+ somata and the number of PV-boutons (Pearson correlation coefficient *r* = −0.83; *p* = 0.039). Finally, we observed that KET mice had fewer PV+ cells expressing Npas4 (*t*_4_ = 6.87; *p* = 0.002—Fig. 2f, h), representing a decrease of about 41% from VEH mice. The number of cells expressing Npas4 outside of PV+ neurons was not affected by neonatal ketamine (*p* > 0.05; Fig. 2g).

### Reduced expression of Npas4 specifically in PV+ cells induces cognitive deficits but does not impact overall activity or emotional behaviors

**Fig. 3:**
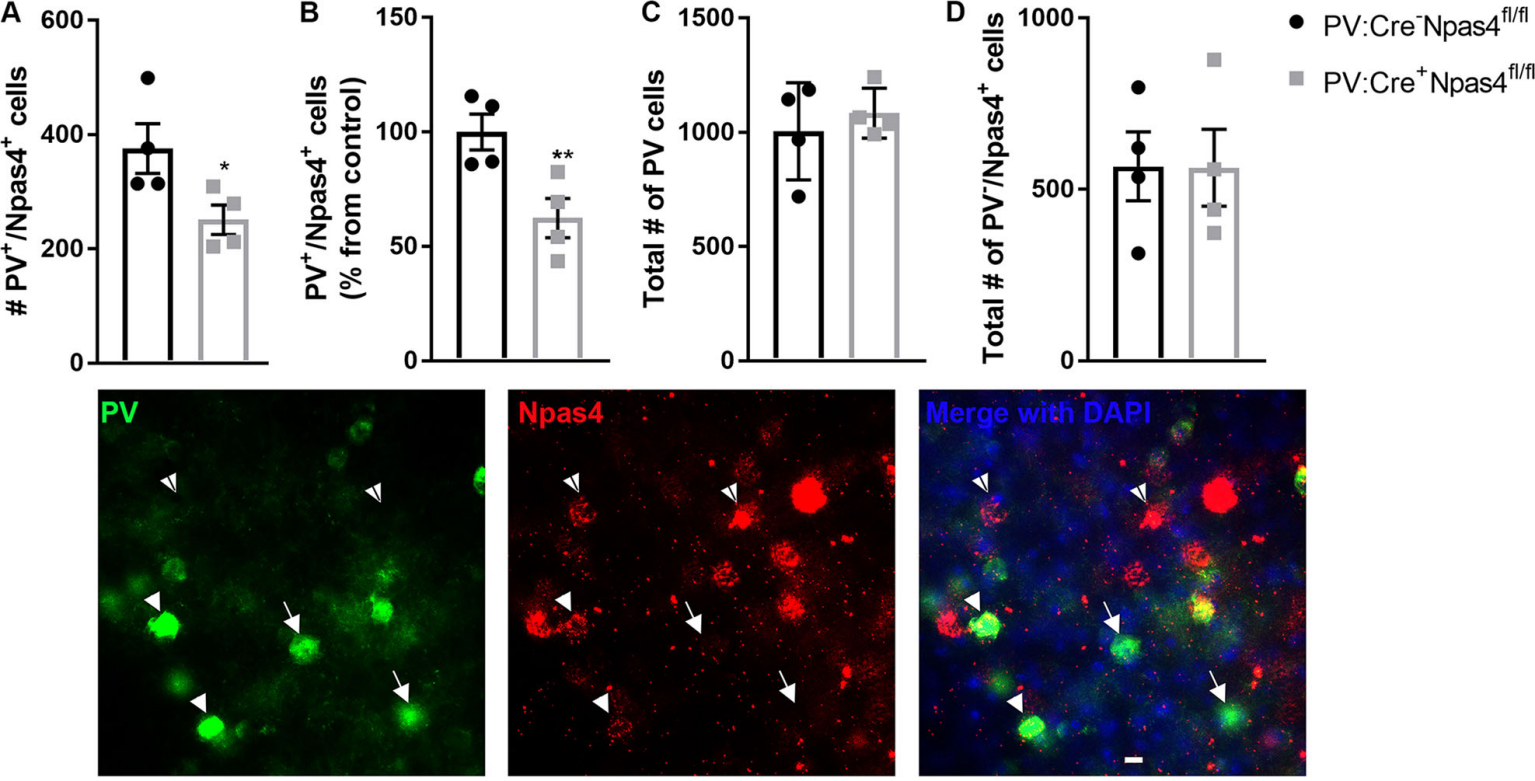
Verification of downregulation of Npas4 in PV+ neurons in the PFC of PV:Cre+Npas4^fl/fl^. Double immunofluorescence staining for PV and Npas4 was assessed with an unbiased stereological quantitative analysis. **a** The number of PV+ neurons expressing Npas4 was significantly lower in PV:Cre^+^Npas4^fl/fl^ than their respective controls (PV:Cre^−^Npas4^fl/fl^—unpaired *t*-test: *t*_6_ = 2.467; *p* = 0.024). **b** This corresponds to a decrease in expression of 38% vs. controls (*t*_6_ = 3.239; *p* = 0.008). **c** The total number of PV+ neurons and (**d**) the number of Npas4-expressing cells that are not PV+ neurons were not different between the two groups of mice (*p* > 0.05). The photomicrographs are representative pictures of immunofluorescence staining for PV (green), Npas4 (red), and DAPI (blue). Full white arrow heads point to a double-stained cell (PV+ neurons expressing Npas4), arrows point toward PV+ neurons not expressing Npas4, and white arrowheads with black stripe point toward Npas4-expressing cells that are not PV+ neurons. ^*^p ≤ 0.05; ^**^p < 0.01; *n* = 3 PFC sections from four mice per genotype

Our goal was to determine the extent to which reduced expression of Npas4 specifically in PV+ neurons would be sufficient to replicate the cognitive and behavioral impairments observed after exposure to neonatal ketamine. Neonatal exposure to ketamine can affect multiple neuronal circuits and neurotransmitter systems that could each contribute to the behavioral impairments observed. We thus meant to isolate Npas4 downregulation in PV+ neurons from all other potential molecular pathways potentially affected by developmental ketamine. We used a Cre-Lox approach to selectively knockdown Npas4 in PV+ neurons. We verified by double-immunofluorescent techniques that PV+ neurons of the prefrontal of PV:Cre^+^Npas4^fl/fl^ mice express significantly less Npas4 than their littermate controls. We observed a significant reduction in the number of PV+ neurons expressing Npas4 in PV:Cre^+^Npas4^fl/fl^ when compared to control PV:Cre^−^Npas4^fl/fl^ animals (38% from control mice; *t*_6_ = 2.467; *p* = 0.024—Fig. 3a, b), without any change in the total number of PV+ neurons or the number of non-PV+ cells expressing Npas4 (*p* > 0.05—Fig. 3c, d).

**Fig. 4:**
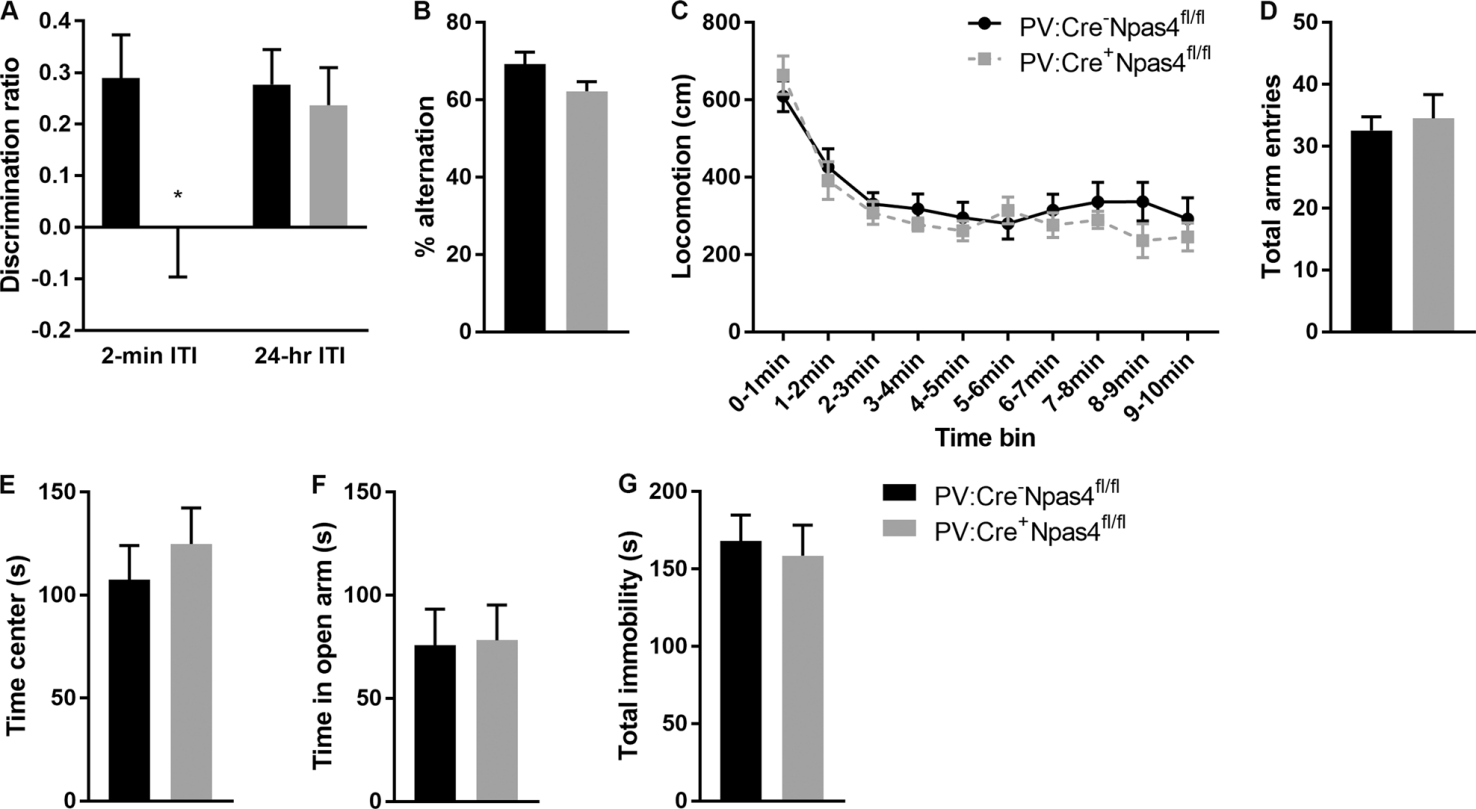
Behavioral phenotyping of mice with downregulation of Npas4 specifically in PV+ neurons (PV:Cre^+^Npas4^fl/fl^ mice) compared to their wild-type controls (PV:Cre^−^Npas4^fl/fl^ mice). **a** Short-term (2-min ITI), but not long-term (24-h ITI), object memory was impaired in PV:Cre^+^Npas4^fl/fl^ mice (*t*_14_ = 2.202; *p* = 0.045). **b** Spontaneous alternation measured in the Y-maze test was not affected by the genotype (*p* > 0.05). Similarly, no genotype effect (*p* > 0.05) was observed for general locomotor activity in (**c**) the open-field arena or (**d**) in the elevated plus maze, for anxiety-like behaviors in (**e**) in the open-field test or (**f**) the elevated plus maze test, and for (**g**) depressive-like behaviors in the forced swim test. ^*^p ≤ 0.05; PV:Cre^+^Npas4^fl/fl^ mice *n* = 8–9; PV:Cre^−^Npas4^fl/fl^ mice; *n* = 7–8

PV:Cre^+^Npas4^fl/fl^ mice exhibited impaired cognitive abilities in the ORT with a 2-min ITI (*t*_14_ = 2.21; *p* = 0.045—Fig. 4a), while no deficits were observed when the ITI was set at 24 h (*p* > 0.05—Fig. 4a) or in the Y maze test (*p* > 0.05—Fig. 4b). No difference in the total time spent sniffing the objects during the learning phase or testing phase of the ORT was noted (*p* > 0.05). PV:Cre^+^Npas4^fl/fl^ mice displayed no change in novelty-induced locomotor activity in the OFT (repeated measure ANOVA per time bin; genotype effect *p* > 0.05; time bin effect *F*_9, 135_ = 22.25; *p* < 0.0001—Fig. 4c), or in the EPM (total arm entries *p* > 0.05—Fig. 4d). Similarly, no genotype effect was observed regarding emotional behaviors (time in center of OFT *p* > 0.05; time spent in the open arm of the EPM *p* > 0.05; total immobility in FST *p* > 0.05—Fig. 4e–g, respectively).

## Discussion

Our findings implicate for the first time the transcription factor Npas4 to prefrontal PV+ neurons dysfunction and cognitive deficits as seen in schizophrenia and other neurodevelopmental disorders. It is well established that a number of neurodevelopmental disorders are characterized by abnormalities within the prefrontal parvalbumin (PV+) system (e.g. refs. ^1,6^). However, the intracellular contributor to PV+ neurons deregulation and associated cognitive deficits remain elusive. Using a developmental mouse model of schizophrenia, we report for the first time a PV+ neuron-specific downregulation of the brain-specific transcription factor Npas4 following neonatal exposure to ketamine. Previous studies reported that developmental alteration of glutamatergic transmission impacts the developmental trajectory of prefrontal PV+ neurons, thereby leading to a dysfunctional PV system in adulthood and to aberrant behaviors^21,25^. Further work demonstrated that blockage of NMDA receptors during early postnatal development induces oxidative stress that contributes to loss of prefrontal PV+ neurons^28^ suggesting a potential mechanism to explain dysfunction of the prefrontal PV system in a rodent model of schizophrenia. Our novel findings reveal that Npas4 could be part of the cascade of molecular events by which oxidative stress leads to PV+ neurons dysfunction in schizophrenia, and thereby to cognitive deficits. Indeed, recent work reported that Npas4 expression in the hippocampus is reduced by oxidative stress^29^. While this remains to be confirmed and verified in the context of a developmental model of schizophrenia, our findings provide a potential novel molecular contributor to PV+ neurons dysfunction induced by developmental NMDA receptor blockade.

Prenatal and early postnatal exposures to NMDA receptors antagonists induce schizophrenia-like molecular and behavioral phenotypes in rodent models^21,26,30,31^. In the current study, we further confirm that developmental blockage of NMDA receptors leads to cognitive deficits. However, in contradiction to previous studies^21^, we observed that KET mice show hyperactivity in the open-field. Differences in testing conditions might contribute to differences in results, especially since we tested mice during their active period under white light conditions that could have created an anxiogenic environment. This is supported by the findings that locomotor activity was significantly increased near the walls in KET mice, but not in the center of the arena. We further report that the behavioral abnormalities induced by ketamine exposure are associated with long-lasting abnormalities within the prefrontal GABAergic system, including decreased number of PV+ cells and reduced expression of GAD67 in the PFC, two molecular markers of the schizophrenia brain. Jeevakumar and Kroener^26^ reported a similar, persistent, PV-specific effect of developmental exposure to ketamine. Unexpectedly, we report higher total level of PV protein in mice exposed to neonatal ketamine. Our analysis of PV positive staining indicates that while we count less PV+ cell bodies in KET mice, we also count more PV-boutons, indicating that the increased level of PV protein could be localized at the level of neuron terminals. Studies in rat models of epileptic seizures have reported similar findings in the hippocampus and suggested a compensatory mechanism to overcome the partial loss of inhibitory function due to reduced number of PV+ neurons^32,33^. Our findings would support this idea since the extent of PV+ somata loss correlates positively with the number of PV-boutons. While further analyses are needed to confirm this observation and its interpretation, the changes we observed in ketamine-exposed mice support previous findings about abnormal functionality of prefrontal PV+ cells in rodent model of schizophrenia, which can contribute to the deficits in cognitive functions we and others observed.

Importantly, we report the novel finding that neonatal ketamine leads to a persistent downregulation of Npas4 expression specifically within prefrontal PV+ neurons. Previous work reported a downregulation of Npas4 in the PFC from postmortem tissue of schizophrenia patients and from mice with prenatal dysregulation of one-carbon metabolism^20^. Our research confirm these findings by employing a different developmental mouse model of schizophrenia, which resulted in a similar reduction in Npas4 expression. Furthermore, we refine these findings by highlighting a cell type-specific alteration of Npas4 expression, which could significantly contribute to dysfunction of PV+ neurons. Npas4 expression in GABAergic neurons promotes the formation of excitatory synapses onto these neurons to facilitate inhibition in an activity-dependent manner^11^. Thus, reduced Npas4 expression within PV+ neurons, induced by developmental exposure to NMDA receptor antagonist, would lead to decreased PV-dependent inhibitory transmission and to increased overall circuit excitation, similar to that observed in schizophrenia^34^. Our current findings support strongly the need to further examine the exact impact of a downregulation of Npas4 expression within PV+ neurons not only at the cellular level using direct measures of PV+ neurons functions (e.g. measures of firing rate), but also at the circuit level to gain novel knowledge on the molecular mechanisms underlying prefrontal abnormalities in schizophrenia.

To determine the extent to which downregulation of Npas4 within PV+ neurons could contribute to the behavioral abnormalities observed in the neonatal exposure ketamine model of schizophrenia we used a genetic approach. Following selective downregulation of Npas4 in PV+ neurons, we observed specific changes in behavioral functions that overlap only slightly with those observed following neonatal ketamine exposure. It is important to note that, contrary to the neonatal exposure to ketamine model, our genetic model involves the downregulation of Npas4 in PV+ neurons during the entire prenatal and postnatal development of the brain. This could lead to compensatory mechanisms that could explain differences in phenotypes. For instance, we were not able to reproduce the hyperactive phenotype that we reported following neonatal exposure to ketamine. On the other side, we observed cognitive deficits similar to those observed in KET mice, specifically in the ORT with a short 2-min ITI. The ORT task assesses visual learning and memory, a cognitive domain impaired in schizophrenia as highlighted by the NIMH sponsored the Measurement and Treatment Research to Improve Cognition in Schizophrenia (MATRICS) initiative^35^. Our findings demonstrate that downregulation of Npas4 in PV+ neurons is sufficient to alter only short-term but not long-term visual memory. Many brain regions contribute to object recognition, including the hippocampus, peri-rhinal cortex and PFC^35,36^ Involvement of these different regions depends on the ITI and other characteristics of the test, such as whether objects location was changes between the learning and testing phases. Here, we not only introduced a novel object during the testing trial, we also moved the location of the familiar object. This task requires the mouse to process information about “what” changed and “where” changes occur. Which brain region is specifically involved in this form of information processing remain unclear. Human studies suggest that the PFC is involved in episodic object recognition^37^. However, lesions of the PFC in rats do not impair novel object recognition^38^. It will be important in future studies to assess pattern of neuronal activity during the 2-min ITI ORT task we used here to identify alterations within specific brain regions after neonatal ketamine and after genetic deletion of Npas4 within PV+ cells. The lack of deficit in the ORT with a long ITI was unexpected. Memory for objects after long delays (as opposed to short delays) have often been associated with the integrity of the hippocampus^39,40^. Our genetic approach allowed us to downregulate Npas4 in a cell type-specific manner, but not in a brain region-specific way. As such, we anticipated that PV:Cre^+^Npas4^fl/fl^ mice would display a lower expression of Npas4 in hippocampal PV+ neurons. Previous studies described hippocampal-dependent cognitive deficits in mice with impaired functions of hippocampal PV+ neurons. For instance, transgenic mice lacking the GluR4 or GluR1 subunit in PV+ neurons display deficits in spatial reference memory^41^. However, others report unaltered reference memory in mice with NR1 ablation from PV+ neurons or with genetic deletion of PV+ neurons from the CA1 region^42,43^. It is possible that even if there is a downregulation of Npas4 in hippocampal PV+ neurons in our transgenic mouse model, the degree of downregulation might not be sufficient to induce severe hippocampal-dependent cognitive dysfunction (for reference, we obtained a 44% reduction in the PFC).

It is important to note that downregulation of Npas4 in PV+ neurons does not lead to hyperactivity as observed after neonatal ketamine exposure, or after global knockdown of Npas4^12,13^. This shows that different mechanisms might underlie changes in different behavioral domains following neonatal exposure to ketamine. While changes in Npas4 expression within PV+ cells could contribute to cognitive impairments, the effects of developmental NMDA receptor hypofunction on the hyperactive phenotype we observed is likely to be independent of Npas4-induced dysfunction of PV+ neurons. Other molecular mechanisms that lead to dysfunction of PV+ neurons in the PFC might contribute to this behavioral abnormality^44–46^, as well as disturbances in other GABAergic subsystems^47,48^ or other neurotransmitter systems.

In conclusion, our data support the idea that the level of expression of Npas4 within PV+ neurons could be a major molecular contributor to the cognitive deficits observed in several neurodevelopmental disorders marked by PV+ neurons dysfunction. A thorough assessment of the effects of Npas4 expression reduction in PV+ neurons, in prefrontal circuit activity, and in cognitive functions is needed. Such an assessment will establish whether Npas4 downregulation leads to similar changes within PV+ neurons and prefrontal circuits to those observed in schizophrenia. This would further implicate the importance of this transcription factor to developmental cognitive pathologies, which could represent a novel therapeutic target to treat the cognitive symptoms seen in schizophrenia that are to date unresponsive to pharmacological approaches.
